# Inflammatory Ratios as Survival Prognostic Factors in Resectable Gastric Adenocarcinoma

**DOI:** 10.3390/diagnostics13111910

**Published:** 2023-05-30

**Authors:** Vlad-Ionuţ Nechita, Nadim Al-Hajjar, Daniel-Corneliu Leucuța, Emil Moiş, Alin Fetti, Mihaela-Ancuţa Nechita, Florin Graur

**Affiliations:** 1 Department of Medical Informatics and Biostatistics, “Iuliu Hațieganu” University of Medicine and Pharmacy, Louis Pasteur Street, No. 6, 400349 Cluj-Napoca, Romania; nechita.vlad@umfcluj.ro; 2 Octavian Fodor Regional Institute of Gastroenterology and Hepatology Cluj-Napoca, Croitorilor Street, No. 19–21, 400162 Cluj-Napoca, Romania; 3 3rd Department of Surgery, “Iuliu Hațieganu” University of Medicine and Pharmacy, Croitorilor Street, No. 19–21, 400162 Cluj-Napoca, Romania; 4 Ion Chiricuță Oncology Institute, Republicii Street, No. 34–36, 400015 Cluj-Napoca, Romania

**Keywords:** inflammatory ratios, gastric adenocarcinoma, surgical resection, survival

## Abstract

Background: The purpose of the study was to assess the relationship between inflammatory biomarkers (NLR—neutrophil-to-lymphocyte ratio, PLR—platelet-to-lymphocyte ratio, LMR—lymphocyte-to-monocyte ratio, SII—systemic immune-inflammation index) and overall survival in gastric cancer patients. Methods: Over a six-year period (2016–2021), we conducted a longitudinal retrospective cohort research on 549 patients with resectable stomach adenocarcinoma. The overall survival was determined using the univariate and multivariate COX proportional hazards models. Results: The age of the cohort varied between 30 and 89 years old, with an average age of 64.85 ± 10.51 years. Four hundred seventy-six patients (86.7%) had R0 resection margins. Eighty-nine (16.21%) subjects received neoadjuvant chemotherapy. Two hundred sixty-two (47.72%) patients died during the follow-up period. The median survival time in the cohort was 390 days. A significantly lower (*p* = 0.029—Logrank test) median survival was observed for R1 resections (355 days) in comparison with R0 resections (395 days). Significant differences in survival were observed regarding tumor differentiation, tumoral (T), and node (N) stage. No differences in survival were observed between the low or high value of inflammatory biomarkers (dichotomized by median value in the sample). In the COX univariate and multivariate regression models, elevated NLR proved an independent prognostic factor for lower overall survival [HR = 1.068, (95% CI 1.011–1.12)]. In this study, the other inflammatory ratios (PLR, LMR, and SII) did not prove as prognostic factors for gastric adenocarcinoma. Conclusions: In resectable gastric adenocarcinoma, elevated NLR before surgery was associated with lower overall survival. PLR, LMR, and SII had no prognostic value for the patient’s survival.

## 1. Introduction

Gastric cancer is a common malignant pathology, the 5th type of cancer according to frequency and the 4th according to cancer-related mortality [1]. This malignancy is about two times more frequent for the male gender [1,2]. The risk factors for gastric cancer are *Helicobacter pylori* infections, smoking, surgical history of partial gastrectomy, Ménétrier’s disease, and atrophic gastritis, along with the male gender previously mentioned [3].

Over 95% of malignant gastric tumors are adenocarcinomas and can be classified according to the anatomic location (proximal or distal gastric cancer) and microscopically according to the histologic type (diffuse or intestinal) [4]. Neoadjuvant chemotherapy (NAC) can improve the prognostic in advanced gastric cancer [4,5]. NAC can eliminate microscopic metastasis and, having a downstaging effect, can increase the rate of R0 resections [6]. Cunningham et al. showed an improved survival rate (36.3%) to five years for patients with stage II and III gastric cancer that received pre-postoperative chemotherapy, in comparison with the group treated only surgically (23%) [7].

Xiang et al. underlined the importance of a multidisciplinary approach in the treatment of gastric cancer. The multidisciplinary treatment group exhibited a higher three-year survival rate (55.6% vs. 46.1%, *p* = 0.005) and reduced mortality in advanced gastric cancer, as indicated by multivariate analysis (HR = 0.49; *p* < 0.001) [8]. Coburn et al. also emphasized that high-volume, specialized centers exhibit lower rates of post-procedural mortality, while centers with less than five cases per year are considered very low-volume centers [9].

Di Carlo et al. compared the laparoscopic approach (39 patients) with the open surgery (53 patients) total gastrectomy for locally advanced gastric adenocarcinoma (stage II and III), in terms of survival, without finding a significant difference at three and five years. Instead, a significant difference was observed for the median number of resected lymph nodes (*p* = 0.031), which was higher for the laparoscopic total gastrectomy, and the recurrence rate (12% for open surgery vs. 5% for laparoscopic intervention, *p* = 0.048). The rate of R0 resection was also not significantly different between procedures [10].

Moon et al. evaluated the rate of recurrence for patients with gastric adenocarcinoma (stage I–IV, without proof of metastasis at the time of the intervention) that benefit from radical gastrectomy (R0) and adjuvant treatment [11]. The majority of recurrences were observed in the first two years. In the first five years after gastrectomy, the recurrence rate appears to be 41%, with peritoneal carcinomatosis in most cases (51.7%). At five-to-ten years after surgery, the recurrence rate was 10.8%, predominantly with distant metastasis (34.6%), and after ten years from gastrectomy, a 2% recurrence rate was described. Some late recurrence (that is uncommon) is also described in the literature [12].

According to Maroufizadeh et al., the patient’s age at presentation, tumor dimension, and stadialisation are independent prognostic factors for gastric cancer survival [13]. After Shiraishi et al., the independent prognostic factors for survival in large gastric adenocarcinoma (higher than 10 cm diameter) can be considered: serosa involvement (T4; OR = 3.06; *p* < 0.01), metastasis in distal extra gastric lymph nodes (OR = 2.13; *p* < 0.05), and liver metastasis (OR = 3.77; *p* < 0.05). For cases with curative-intent surgery advanced stage (III and IV), the presence of extra gastric lymph nodes and hemoglobin level < 10 g/dL proved to be independent prognostic factors for patients’ survival in the multivariate COX proportional hazards model [14].

The inflammatory ratios were also evaluated by some authors for patients with gastric cancer. Kim et al., in a meta-analysis of 41 studies published between 2007 and 2020 that summed 18.348 participants, suggested that a high NLR value was positively correlated with a lower overall survival for gastric cancer patients, with moderate heterogeneity (the inconsistency index, I^2^ = 50.79%, *p* = 0.002). The combined HR proved a poor prognosis for patients with increased NLR [HR = 1.61, 95% CI (1.45–1.78), *p* < 0.001] than for those with lower NLR values in gastric cancer [15].

In the study of Lian et al., the survival of patients with resectable gastric cancer was evaluated according to the values of NLR and PLR. Significantly better overall survival was observed for the patients with lower values of these biomarkers. Furthermore, higher values of the biomarkers were related to more advanced tumors: higher T stage and higher N stage [16]. Patients with stomach cancer had shorter overall survival rates when their lymphocyte-to-monocyte ratio (LMR) was lower [17,18].

SII (Systemic immune-inflammation index) proved to have a significant prognostic value for the first year of survival in patients with gastric cancer. Lower values were associated with better survival [19]. Fu et al. evaluated the prognostic value of SII for patients with gastric cancer over 11 studies that were included in the meta-analysis. The cut-off values varied between 320 and 802 in different studies. Higher SII value was significantly associated with decreased OS [pooled HR = 1.53, 95% CI (1.27–1.83)], in accordance with eight of the evaluated studies [20].

We aimed to evaluate the prognostic value of the inflammatory biomarkers (NLR, PLR, LMR, and SII) at the presentation in a surgery department for overall survival in patients with resectable gastric adenocarcinoma from the Romanian population in a large longitudinal cohort study.

## 2. Materials and Methods

### 2.1. Study Design

We conducted a retrospective cohort study at the Regional Institute of Gastroenterology and Hepatology ‘Prof. Dr. Octavian Fodor’ in Cluj-Napoca, Romania. The study included patients with resectable gastric adenocarcinoma who underwent curative-intent resection. The available medical charts were consulted for all the patients with gastrectomy between January 2016 to December 2021.

### 2.2. Participants

The study enrolled patients who had confirmed pathology of gastric adenocarcinoma and underwent either total or subtotal gastrectomy with curative intent. Only patients undergoing their initial surgery were included. Patients that refused surgery, with metastasis, advanced tumor without resection, with only palliative surgical procedures, or patients with a pathology result different from adenocarcinoma (GIST, neuroendocrine tumor, lymphoma, benign lesions) or synchronous tumors were excluded.

The pathology result served as the gold standard for diagnosis and staging. For cases deemed unresectable, diagnosis and staging were assessed using medical imaging techniques (ultrasonography, computer tomography, magnetic resonance) or confirmed during laparotomy with metastasis biopsy.

### 2.3. Variables, Data Collection, and Follow-Up

The variables collected for this study included general patient information such as age, gender, setting, and history of previous neoadjuvant treatment. For tumor description, data from the pathology report were used: tumor dimension (mm), tumor invasion (T stage), lymph node invasion (N stage), information about the microscopic invasion (vascular and perineural), grading, Lauren type, and resection margin. The surgical protocol provided information on the type of gastrectomy, invasion, and anastomosis, which were collected for analysis. During the follow-up period, survival information was obtained. The primary endpoint of the study was the overall survival, defined as the time in days from surgery to the patient death. Survival data were obtained from population records in Cluj-Napoca up to 7 October 2022. Before any treatment in the surgery department, preoperative blood samples were collected at the patient’s admission. The absolute counts for neutrophils (10^3^/μL), platelets (10^3^/μL), lymphocytes (10^3^/μL), and monocytes (10^3^/μL) were recorded. To compute the specific ratios, we divided the absolute values. For SII (Systemic Immune-Inflammation Index), the product between platelets and neutrophils was divided by the lymphocyte value.

### 2.4. Statistical Analysis

To present qualitative data, we used absolute values and percentages; for quantitative data, the average and standard deviation, respectively, medians, and interquartile range based on the distribution of values. The survival between the groups was compared with the Log-rank test and represented by Kaplan–Meyer curves. Variables with potential prognostic significance for survival were evaluated in univariate COX regressions. The NLR, PLR, LMR, and SII, were evaluated as continuous values or dichotomized by the median value of the sample. The multivariate COX regression models were built for the variables above, adjusted for age, tumor dimension, T stage, N stage, grading, neoadjuvant chemotherapy, positive resection margin and microscopic invasion. The proportional hazard assumption and the multicollinearity assumption were checked for the models. For interpretation, we presented the HR (hazard ratio) with the corresponding 95% confidence interval and the *p*-value. A significant *p*-value was considered below 0.05. To perform the statistics, we used R version 4.0.5 (R Foundation for Statistical Computing, Vienna, Austria) [21].

We hypothesized that the inflammatory ratios (NLR, PLR, LMR, and SII) collected at the patient’s presentation have potential prognostic value for survival in patients with resectable gastric adenocarcinoma.

### 2.5. Ethical Statement

This study received Ethical Committee approval from the University of Medicine and Pharmacy (“Iuliu Hațieganu” Ethical Committee, number 265/30.06.2021) and from the Hospital (Regional Institute of Gastroenterology and Hepatology Ethical Committee, number 2480/23.02.2022).

## 3. Results

We evaluated 549 patients who underwent curative-intent resection for gastric adenocarcinoma between 4 January 2016 and 22 December 2021. Four hundred seventy-six patients (86.7%) had free resection margins (R0) after the intervention. The rate of R1 resection was 13.29% (73/549). The major characteristics of the cohort according to the resection margin are presented in Table 1. The age of the included subjects was between 30 and 89 years old, with an average of 64.85 ± 10.51 years. The male-to-female ratio was 2.14. Eighty-nine (16.21%) subjects received neoadjuvant chemotherapy before admission to surgery.

**Figure 1:**
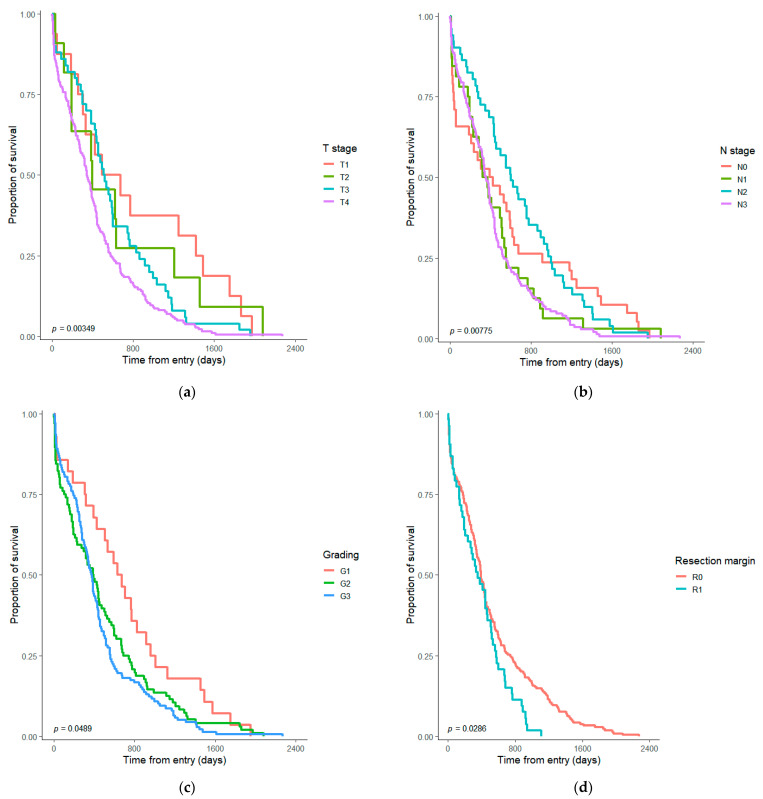
Kaplan–Meyer graph with survival differences in resectable gastric adenocarcinoma according to (**a**) tumor invasion (T); (**b**) lymph node involvement (N); (**c**) tumor differentiation (grading); and (**d**) resection margin.

Out of the patients, 246 (44.81%) had corporeal gastric tumors, 226 (41.16%) had antral tumors, and 77 (14.02%) had cardia tumors. No statistically significant difference was observed in survival (*p* = 0.39—Logrank test) based on the tumor localization in resectable gastric adenocarcinoma. Among the patients, 238 (43.35%) underwent total gastrectomy, while 311 (56.64%) underwent subtotal gastrectomy. One hundred eighteen (21.49%) patients benefit from mechanical anastomosis. During the follow-up period, 262 patients (47.72%) passed away. The median survival time in the cohort was 390 days, 95% CI (343–440). A statistically significant difference (*p* = 0.029—Logrank test) was observed between median survival time in the group with R0 resections [392, 95% CI (344–441) days] in comparison with R1 resections [355, 95% CI (237–508) days] (Figure 1).

Two hundred and eighty-nine (52.6%) patients had T4 tumors at their presentation, and sixty-three (11.47%) patients presented resectable macroscopic invasion in the nearby organs at the time of surgery. A significant difference was observed in survival according to the T stage (*p* = 0.003—Logrank test) and a significantly lower median survival (*p* < 0.001—Logrank test) for patients with macroscopic invasion in other organs (252 days) in comparison to those without invasion (425 days) (Figure 1). Regarding the presence of microscopic invasion, the pathology report showed 299 cases (54.46%) with perineural invasion and 183 patients (33.33%) with microvascular invasion. No statistically significant differences were observed for the presence of microscopic perineural invasion (*p* = 0.9—Logrank test), but a significantly lower median survival (*p* < 0.001—Logrank test) for the microvascular invasion (338 days vs. 452 days). The difference in survival was significant according to the N stage (*p* = 0.008—Logrank test) and tumor grading (*p* = 0.048—Logrank test), but without significance for the Lauren type (*p* = 0.25—Logrank test) (Figure 1).

**Figure 2:**
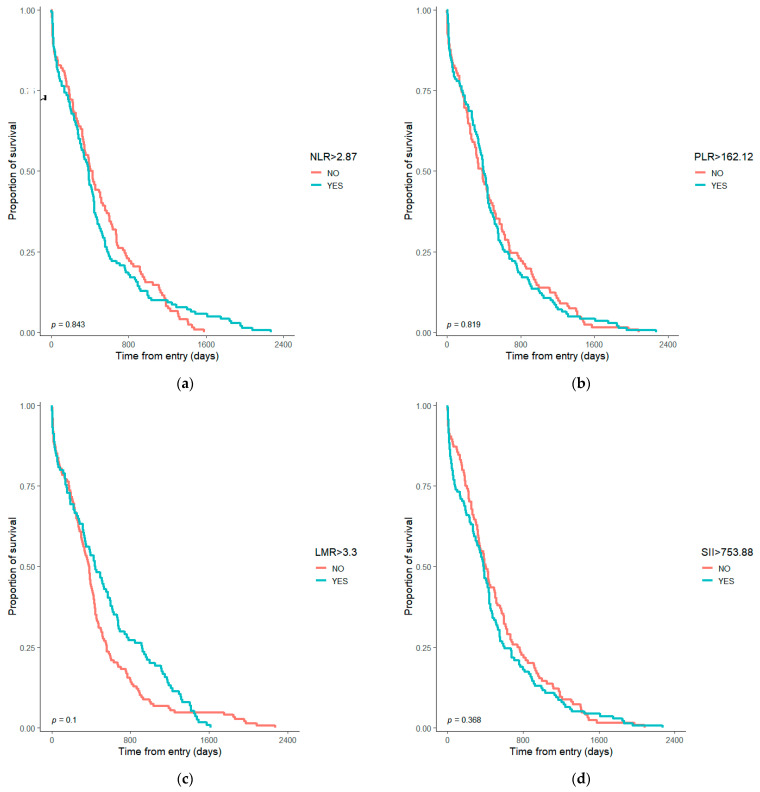
Kaplan–Meyer survival graph with the differences for resectable gastric adenocarcinoma according to inflammatory ratios median values: (**a**) Survival considering NLR > 2.87 (median cohort value); (**b**) Survival considering PLR > 162.12 (median cohort value); (**c**) Survival considering LMR > 3.3 (median cohort value); (**d**) Survival considering SII > 753.88 (median cohort value); NLR—Neutrophil-to-lymphocyte ratio; PLR—Platelet-to-lymphocyte ratio; LMR—Lymphocyte-to-monocyte ratio; SII—Systemic immune-inflammation index.

No statistically significant differences in survival were observed when NLR, PLR, LMR, and SII were above or below the median value (Figure 2).

We evaluated the relationship with the overall survival of the inflammatory ratios for patients with resectable gastric adenocarcinoma (Table 2). In the COX univariate model, it emerged that macroscopic invasion [(*p* < 0.001, HR = 2.02 95% CI (1.47–2.79)], higher tumor stage [(*p* = 0.004, HR = 2.15 95% CI (1.27–3.64) for T4], tumor dimension [(*p* = 0.002, HR = 1.006 95% CI (1.002–1.01)], higher N stage [(*p* = 0.02, HR = 1.5 95% CI (1.04–2.17 for N3)], positive resection margins [(*p* = 0.029, HR = 1.4 95% CI (1.035–1.92)], and bad differentiated tumors [(*p* = 0.04, HR = 1.66 95% CI (1.10–2.51) for G3 tumor] were associated with poor survival. No significant associations in univariate models were observed for age [(*p* = 0.071, HR = 1.011 95% CI (0.99–1.022)], neoadjuvant chemotherapy [(*p* = 0.11, HR = 0.75 95% CI (0.52–1.072)], and Lauren type [(*p* = 0.3, HR = 1.057 95% CI (0.75–1.47)].

At the presentation, only the absolute value of NLR among the inflammatory ratios was significantly associated with a low chance of survival in the univariate analysis (Table 2). The other inflammatory ratios’ absolute values, or dichotomized according to the median value of the sample, were not significant in the univariate models. In multivariate analysis, adjusted for macroscopic and microscopic tumor characteristics, age, resection margin, and neoadjuvant treatment, only the NLR absolute value at admission continued to be statistically significant (Table 2).

## 4. Discussion

Our study evaluated the inflammatory biomarkers as prognostic factors for the long-term survival of a large cohort of gastric cancer patients, a frequently diagnosed malignant pathology and an important cause of death worldwide [1]. The higher NLR values were associated with poor survival even after adjustment for multiple confounders, while the other inflammatory makers failed to be associated with survival.

Upon admission for surgery, more than half of the patients (52.6%) presented with T4 tumors, indicating a late-stage diagnosis where surgical options may be limited. The rate of R1 resections was 13.29%, with approximately 85% of the cases occurring in the T4 tumor stage (Table 1). Additionally, over 60% of the cases were poorly differentiated (Table 1). In the literature, the absence of radical resections is reported to range from 1.8% to 9% for gastric adenocarcinoma and more than 10% for the tumors of gastroesophageal junction [22]. A slightly higher R1 rate can be explained in our sample by the late presentation, with advanced disease at the patient’s arrival to surgery. Ridwelski et al. also noted that the rate of R1 resections increases with tumor size (>5 cm), tumor stage (T34), N stage, and poor differentiation [22]. Accordingly, Shiraishi et al. observed a poor prognosis for patients with extragastric lymph node involvement with less than a 6% survival rate at five years, regardless of the surgical management [14]. On patients with large gastric adenocarcinoma (>10 cm diameter), they described a median survival of 15 months and a 22% survival rate at five years. For patients with curative gastrectomy, the 5-year survival rate was 33%, and the median survival 27 months [14]. Tumor dimension proved significant for the overall survival also in our sample [(*p* = 0.002, HR = 1.006 95% CI (1.002–1.01)] in the univariate model and was considered as a covariate in the multivariate analysis.

Stiekema et al. studied the prognostic value of R1 resection for recurrence-free survival over a consecutive series of 110 patients with resected gastric cancer that followed radiochemotherapy. The rate of R1 was 27% (30/110). No statistically significant difference was observed between R0 and R1 while the patients received adjuvant treatment. Otherwise, the T and N stages presented significance for survival in the multivariate analysis [23]. In our retrospective cohort, only the information about neoadjuvant therapy before surgery was available for data collection and was considered in the multivariate model. For patients with advanced diffuse-type gastric cancer, the T stage (HR = 4.5, *p* < 0.001) and positive resection margins (HR = 3.5, *p* = 0.001) proved independent prognostic factors to predict overall survival [24]. A significantly higher (*p* = 0.029—Logrank test) median survival time (392 days) was also observed in our sample for the R0 group in comparison with the R1 resection (355 days). The differences were also observed for the T and N stages in our study, according to the literature mentioned above (Figure 1).

More than 80% of the tumors had antral or corporeal localization, and only 14% were cardia tumors in our study, but without a significant difference in survival according to the tumor localization (*p* = 0.39—Logrank test). According to the meta-analysis by Xue et al., the first-year survival rate for proximal gastric cancer was lower in comparison with distal gastric cancer. The differences were still significant at three and five years, with a lower survival rate for proximal gastric cancer only in the Eastern countries, while for Western countries, the differences after the first year were not significant anymore [25]. Based on Shiraishi et al. study, the tumor location (proximal or distal) proved without statistical significance in the univariate COX proportional hazard model [14].

Regarding the microscopic tumor characteristics, a significant difference in survival was observed according to the tumor differentiation (Figure 1c). In the univariate COX proportional hazard model of Shirashi et al., the presence of microscopic invasion was found to be statistically non-significant for the overall survival [14]. For our patients, the perineural invasion did not make significant differences on the survival, but the presence of microscopic vascular invasion was statistically significant for the lower overall survival (*p* < 0.001—Logrank test).

Nakamura et al. evaluated the NLR as a prognostic factor in stage IV gastric cancer, suggesting that patients with lower values of the indicator have a better response to neoadjuvant chemotherapy and improved conversion to surgery and curative resection [26]. Improved survival for patients with lower NLR after neoadjuvant treatment was also described by Liu et al. [27]. A multicentric study over 490 subjects with gastric cancer, with 309 gastrectomies, suggested that NLR values higher than 5 [HR = 2.24 (95% CI: 1.72–2.92), *p*  <  0.001] and PLR higher than 350 [HR = 2.33 (95% CI: 1.73–3.13), *p*  <  0.001] are associated with lower overall survival [28]. Peng et al., in a meta-analysis of 17 studies and 3499 patients, also suggested that a higher PLR is related to lower survival [pooled HR = 1.43 (95% CI: 1.25–1.64), *p* < 0.001] and poor response to chemotherapy [29].

The inflammatory ratios dichotomized by median values of the sample proved without statistical significance for survival (Figure 2). In univariate COX models, only NLR was statistically significant [HR = 1.064, 95% CI (1.01–1.121), *p* = 0.02] (Table 2). Alongside the other variables proved to influence the survival in the univariate models and in the studies mentioned above, we mention that age had a wide range for the studied sample between 30 and 89 years old, so the adjustment for age in the multivariate model was mandatory, even if in the univariate did not prove significance. We have to mention that according to Worldometer, the life expectancy in Romania is below the maximum age in our sample (79.9 years for females and 73.1 years for males) [30].

Two multivariate COX regression models were built (with and without microscopic invasion) (Table 2), and for both of them, the absolute value of NLR proved an independent prognostic factor for overall survival, following the trend from the literature [15,16,26,27]. The other inflammatory ratios did not prove to be significant in the prognostic model, despite the concordance suggested in other studies [17,18,19,20,29]. All the adjustments were made for the most important macroscopically and microscopically tumor characteristics. T and N classes were considered individually (not combined as the TNM stage) due to the fact that patients candidates for resection were without metastasis and a large number of subjects and deaths allowed to consider all the relevant covariates. Postoperative chemotherapy was not documented properly for the retrospective study, but the neoadjuvant treatment was considered to improve the model.

Despite a large number of subjects, the selection method, and a good management of confounders, here are some limits we can mention for our study. The concomitant inflammatory conditions or medications were not evaluated; the single-center experience and retrospective data collection can be improved in further research. NLR can be an adjuvant costless prognostic factor for resectable gastric adenocarcinoma. To thoroughly evaluate the predictive significance of inflammatory ratios for gastric cancer, more research with prospective designs, long-term follow-up, and properly corrected confounding variables are required.

## 5. Conclusions

Higher preoperative NLR values can be considered an independent prognostic factor for poor overall survival in resectable gastric adenocarcinoma. PLR, LMR, and SII did not prove significant statistically in the COX multivariate analysis.

## Figures and Tables

**Table 1:** The main characteristics of the individuals and the inflammatory rapports based on the resection margin.

	All Subjects (*n* = 549)	Free-Margins Resection (R0) (*n* = 476)	Positive Margins Resection (R1) (*n* = 73)
Age (years) ^a^	64.85 ± 10.51	65.15 ± 10.36	62.89 ± 11.30
Male gender ^b^	374 (68.12%)	322 (67.65%)	52 (71.23%)
Urban setting ^b^	319 (58.11%)	278 (58.40%)	41 (56.16%)
Lauren type ^b^			
intestinal	268 (48.82%)	244 (51.26%)	24 (32.87%)
mixed	180 (32.78%)	148 (31.09%)	32 (43.83%)
diffuse	101 (18.39%)	84 (40.47%)	17 (23.28%)
Tumor stage (T) ^b^			
T1	72 (13.1%)	71 (14.92%)	1 (1.36%)
T2	57 (10.4%)	55 (11.55%)	2 (2.74%)
T3	131 (23.9%)	123 (25.84%)	8 (10.95%)
T4	289 (52.6%)	227 (47.69%)	62 (84.93%)
Tumor dimension ^a^	51.02 ± 29.66	48.66 ± 28.43	66.39 ± 32.97
Grading ^b^			
G1	88 (16.03%)	82 (17.23%)	6 (8.22%)
G2	209 (38.07%)	186 (39.07%)	23 (31.51%)
G3	252 (45.9%)	208 (43.69%)	44 (60.27%)
Node stage (N) ^b^			
N0	158 (28.78%)	152 (31.93%)	6 (8.22%)
N1 (1–2 regional lymph nodes)	85 (15.48%)	77 (16.17%)	8 (10.96%)
N2 (3–6 regional lymph nodes)	110 (20.03%)	100 (21.00%)	10 (13.69%)
N3 (more than 6 regional lymph nodes)	193 (35.15%)	145 (30.46%)	48 (65.75%)
Event (death) ^b^	262 (47.72%)	209 (43.90%)	53 (72.60%)
Median survival time (days)	390 (159–676)	392 (178–742)	355 (135–571)
NLR at admission	2.87 (2.05–4)	2.84 (1.99–3.94)	3.15 (2.31–4.49)
PLR at admission	162.12 (114.87–217.02)	156.86 (114.49–214.26)	182.58 (122.04–252.44)
LMR at admission	3.3 (2.44–4.35)	3.35 (2.46–4.39)	2.87 (2.15–4.11)
SII at admission	753.88 (466.12–1232.64)	737.96 (447.23–1224.19)	900.11 (550.02–1419.75)

Results are presented as median and IQR—interquartile range; ^a^ Tumor dimension is resented as mean and standard deviation. ^b^ Presented as the absolute value and percentages; NLR—Neutrophil-to-lymphocyte ratio; PLR—Platelet-to-lymphocyte ratio; LMR—Lymphocyte-to-monocyte ratio; SII—Systemic immune-inflammation index.

**Table 2:** Cox proportional hazard regressions, adjusted for age, grading, neoadjuvant treatment, tumor dimensions, resection margin, TN stage and microscopic invasion in resectable gastric adenocarcinoma.

	HR Unadjusted	(95% CI)	*p*	HR Adjusted *	(95% CI)	*p*	HR Adjusted **	(95% CI)	*p*
NLR	1.064	(1.01–1.121)	0.0204	1.068	(1.011–1.12)	0.018	1.063	(1.0038–1.12)	0.036
PLR	1.001	(0.99–1.002)	0.481	0.99	(0.99–1.0013)	0.89	1.002	(0.998–1.0012)	0.77
LMR	0.94	(0.88–1.014)	0.113	0.97	(0.91–1.044)	0.44	0.97	(0.91–1.05)	0.54
SII	1.0001	(0.999–1.0002)	0.303	1.0001	(0.99–1.0002)	0.4	1	(0.999–1.0002)	0.68
NLR ≥ median	1.025	(0.79–1.31)	0.85	1.018	(0.78–1.32)	0.89	0.96	(0.74–1.26)	0.81
PLR ≥ median	1.03	(0.81–1.31)	0.82	0.98	(0.75–1.27)	0.89	0.971	(0.741–1.271)	0.83
LMR ≥ median	0.81	(0.63–1.042)	0.1	0.86	(0.66–1.12)	0.25	0.83	(0.636–1.087)	0.177
SII ≥ median	1.12	(0.87–1.43)	0.37	1.041	(0.8–1.35)	0.75	0.998	(0.766–1.3)	0.99

HR—Hazard ratio; CI—Confidence interval; NLR—Neutrophil-to-lymphocyte ratio; PLR—Platelet-to-lymphocyte ratio; LMR—Lymphocyte-to-monocyte ratio; SII—Systemic immune-inflammation index; Median values were 2.87 for NLR, 162.12 for PLR, 3.3 for LMR, and 753.88 for SII. * Adjusted for age, grading, neoadjuvant chemotherapy, N stage, T stage, tumor dimension (mm), and resection margin (R1). ** Adjusted for the criteria mentioned above and microscopic invasion (vascular or perineural).

## Data Availability

Not applicable.

## References

[1] Sung H., Ferlay J., Siegel R.L., Laversanne M., Soerjomataram I., Jemal A., Bray F. (2021). Global Cancer Statistics 2020: GLOBOCAN Estimates of Incidence and Mortality Worldwide for 36 Cancers in 185 Countries. CA Cancer J. Clin..

[2] Rawla P., Barsouk A. (2019). Epidemiology of gastric cancer: Global trends, risk factors and prevention. Prz. Gastroenterol..

[3] Forman D., Burley V.J. (2006). Gastric Cancer: Global Pattern of the Disease and an Overview of Environmental Risk Factors. Best Pract. Res. Clin. Gastroenterol..

[4] Ajani J.A., D’Amico T.A., Bentrem D.J., Chao J., Cooke D., Corvera C., Das P., Enzinger P.C., Enzler T., Fanta P. (2022). Gastric Cancer, Version 2.2022, NCCN Clinical Practice Guidelines in Oncology. J. Natl. Compr. Canc. Netw..

[5] Smyth E.C., Verheij M., Allum W., Cunningham D., Cervantes A., Arnold D. (2016). Gastric Cancer: ESMO Clinical Practice Guidelines for Diagnosis, Treatment and Follow-Up. Ann. Oncol..

[6] Smyth E.C., Nilsson M., Grabsch H.I., van Grieken N.C., Lordick F. (2020). Gastric Cancer. Lancet.

[7] Cunningham D., Allum W.H., Stenning S.P., Thompson J.N., Van de Velde C.J.H., Nicolson M., Scarffe J.H., Lofts F.J., Falk S.J., Iveson T.J. (2006). Perioperative Chemotherapy versus Surgery Alone for Resectable Gastroesophageal Cancer. N. Engl. J. Med..

[8] Xiang Y.-Y., Deng C.-C., Liu H.-Y., Kuo Z.-C., Zhang C.-H., He Y.-L. (2022). The Prognostic Effect of Multidisciplinary Team Intervention in Patients with Advanced Gastric Cancer. Curr. Oncol..

[9] Coburn N., Cosby R., Klein L., Knight G., Malthaner R., Mamazza J., Mercer C.D., Ringash J. (2017). Staging and Surgical Approaches in Gastric Cancer: A Clinical Practice Guideline. Curr. Oncol..

[10] Di Carlo S., Siragusa L., Fassari A., Fiori E., La Rovere F., Izzo P., Usai V., Cavallaro G., Franceschilli M., Dhimolea S. (2022). Laparoscopic versus Open Total Gastrectomy for Locally Advanced Gastric Cancer: Short and Long-Term Results. Curr. Oncol..

[11] Moon Y.W., Jeung H.C., Rha S.Y., Yoo N.C., Roh J.K., Noh S.H., Kim B.S., Chung H.C. (2007). Changing patterns of prognosticators during 15-year follow-up of advanced gastric cancer after radical gastrectomy and adjuvant chemotherapy: A 15-year follow-up study at a single Korean institute. Ann. Surg. Oncol..

[12] Blanchette P., Lipton J.H., Barth D., Mackay H. (2013). Case Report of Very Late Gastric Cancer Recurrence. Curr. Oncol..

[13] Maroufizadeh S., Hajizadeh E., Baghestani A.R., Fatemi S.R. (2011). Multivariate analysis of prognostic factors in gastric cancer patients using additive hazards regression models. Asian Pac. J. Cancer Prev..

[14] Shiraishi N., Sato K., Yasuda K., Inomata M., Kitano S. (2007). Multivariate Prognostic Study on Large Gastric Cancer. J. Surg. Oncol..

[15] Kim M.R., Kim A.S., Choi H.I., Jung J.H., Park J.Y., Ko H.J. (2020). Inflammatory Markers for Predicting Overall Survival in Gastric Cancer Patients: A Systematic Review and Meta-Analysis. PLoS ONE.

[16] Lian L., Xia Y.-Y., Zhou C., Shen X.-M., Li X.-L., Han S.-G., Zheng Y., Mao Z.-Q., Gong F.-R., Wu M.-Y. (2015). Application of Platelet/Lymphocyte and Neutrophil/Lymphocyte Ratios in Early Diagnosis and Prognostic Prediction in Patients with Resectable Gastric Cancer. Cancer Biomark..

[17] Pan Y.-C., Jia Z.-F., Cao D.-H., Wu Y.-H., Jiang J., Wen S.-M., Zhao D., Zhang S.-L., Cao X.-Y. (2018). Preoperative Lymphocyteto-Monocyte Ratio (LMR) Could Independently Predict Overall Survival of Resectable Gastric Cancer Patients. Medicine.

[18] Ma J., Liu Q. (2018). Clinicopathological and Prognostic Significance of Lymphocyte to Monocyte Ratio in Patients with Gastric Cancer: A Meta-Analysis. Int. J. Surg..

[19] Li C., Tian W., Zhao F., Li M., Ye Q., Wei Y., Li T., Xie K. (2018). Systemic Immune-Inflammation Index, SII, for Prognosis of Elderly Patients with Newly Diagnosed Tumors. Oncotarget.

[20] Fu S., Yan J., Tan Y., Liu D. (2021). Prognostic Value of Systemic Immune-Inflammatory Index in Survival Outcome in Gastric Cancer: A Meta-Analysis. J. Gastrointest. Oncol..

[21] R Core Team (2019). R: A Language and Environment for Statistical Computing.

[22] Ridwelski K., Fahlke J., Huß M., Otto R., Wolff S. (2017). R1 resection for gastric carcinoma. Chirurg.

[23] Stiekema J., Trip A.K., Jansen E.P.M., Boot H., Cats A., Ponz O.B., Verheij M., van Sandick J.W. (2014). The Prognostic Significance of an R1 Resection in Gastric Cancer Patients Treated with Adjuvant Chemoradiotherapy. Ann. Surg. Oncol..

[24] Gaspar-Figueiredo S., Joliat G.-R., Borgstein A.B.J., Van Berge Henegouwen M.I., Brunel C., Demartines N., Allemann P., Schäfer M. (2022). Impact of Positive Resection Margins (R1) on Long-Term Survival of Patients with Advanced Diffuse Type Gastric Cancer. Br. J. Surg..

[25] Xue J., Yang H., Huang S., Zhou T., Zhang X., Zu G. (2021). Comparison of the Overall Survival of Proximal and Distal Gastric Cancer after Gastrectomy: A Systematic Review and Meta-Analysis. World J. Surg. Oncol..

[26] Nakamura N., Kinami S., Tomita Y., Miyata T., Fujita H., Takamura H., Ueda N., Kosaka T. (2020). The Neutrophil/Lymphocyte Ratio as a Predictor of Successful Conversion Surgery for Stage IV Gastric Cancer: A Retrospective Study. BMC Cancer.

[27] Liu Z., Liang Y., Tang X., Qu H. (2021). Decrease in Blood Neutrophil-to-Lymphocyte Ratio Indicates Better Survival After Neoadjuvant Chemotherapy in Patients With Advanced Gastric Cancer. Front. Surg..

[28] Ramos-Esquivel A., Brenes D., Cordero E., Alpizar-Alpizar W. (2018). Neutrophil to Lymphocyte Ratio and Platelet to Lymphocyte Ratio Are Independent Prognostic Factors for Overall Survival in Hispanic Patients with Gastric Adenocarcinoma. Ann. Oncol..

[29] Peng X., Zeng W., Tang B., He A., Zhang M., Luo R. (2022). Utility of Pretreatment Blood Platelet-To-Lymphocyte Ratio in Prediction of Clinical Outcomes and Chemosensitivity in Patients with Advanced Gastric Cancer: A Meta-Analysis. Med. Sci. Monit. Int. Med. J. Exp. Clin. Res..

[30] Romania Demographics 2020 (Population, Age, Sex, Trends)—Worldomete. https://www.worldometers.info/demographics/romania-demographics/#median-age.

